# Physiological Responses and Evaluation of Effects of BMI, Smoking and Drinking in High Altitude Acclimatization: A Cohort Study in Chinese Han Young Males

**DOI:** 10.1371/journal.pone.0079346

**Published:** 2013-11-08

**Authors:** Qian-qian Peng, Zhuoma Basang, Chao-ying Cui, Lei Li, Ji Qian, Quzhen Gesang, La Yang, Zong La, Yang De, Puchi Dawa, Ni Qu, Qu Suo, Zhen Dan, Duoji Xiao, Xiao-feng Wang, Li Jin

**Affiliations:** 1 MOE Key Laboratory of Contemporary Anthropology, School of Life Sciences and Institutes of Biomedical Sciences, Fudan University, Shanghai, China; 2 High Altitude Health Science Research Center, Tibet University, Tibet Autonomous Region, China; 3 CMC Institute of Health Sciences, Taizhou, Jiangsu Province, China; The Centre for Research and Technology, Hellas, Greece

## Abstract

High altitude acclimatization is a series of physiological responses taking places when subjects go to altitude. Many factors could influence these processes, such as altitude, ascending speed and individual characteristics. In this study, based on a repeated measurement design of three sequential measurements at baseline, acute phase and chronic phase, we evaluated the effect of BMI, smoking and drinking on a number of physiological responses in high altitude acclimatization by using mixed model and partial least square path model on a sample of 755 Han Chinese young males. We found that subjects with higher BMI responses were reluctant to hypoxia. The effect of smoking was not significant at acute phase. But at chronic phase, red blood cell volume increased less while respiratory function increased more for smoking subjects compared with nonsmokers. For drinking subjects, red blood cell volume increased less than nondrinkers at both acute and chronic phases, while blood pressures increased more than nondrinkers at acute phase and respiratory function, red blood cell volume and oxygen saturation increased more than nondrinkers at chronic phase. The heavy and long-term effect of smoking, drinking and other factors in high altitude acclimatization needed to be further studied.

## Introduction

High altitude acclimatization is a series of physiological processes which takes place when subjects go to altitude [1]–[2]. At 12,000 feet (3,658 meters), the barometric pressure is only 483 mmHg, so there are roughly 40% fewer oxygen molecules per breath [3]. Decreased oxygen availability in the ambient air is the only environmental stress unique to high terrestrial altitudes. It lowers the oxygen supply to body tissues [4]. The body must adjust to having less oxygen, for which a series of physiological responses take place, including ventilation function, cardiac function, oxygen delivery function, hematology, muscle structure and metabolism, oxygen consumption and so on [5]. The tissues of the body gradually adjusted themselves to defend against the fall in oxygen partial pressure and mitigate its effect to a remarkable degree.

Lowland residents rapidly ascending to high altitude (12,000–18,000 feet or 3,658–5,487 meters) or extreme altitude (18,000+ feet or 5,500+ meters) are at risk of developing high altitude illness [6], such as acute mountain sickness, high altitude pulmonary edema, high altitude cerebral edema, and substantial impairment of physical or cognitive work performance [7]. High altitude acclimatization is therefore the best strategy for prevention of acute mountain sickness [8] as well as other high altitude diseases, and allows people to achieve the maximum physical and cognitive work performances possible at altitude.

However, the process of high altitude acclimatization varied among different individuals, as there are many factors influencing its outcomes. Sherpa et al. (2010) and Mandal et al. (2011) found that in altitude residents body mass index (BMI) indicated an inverse relationship with altitude [9]–[10]. There is scarce research on investigating the relationship between BMI and high altitude acclimatization, but smoking and drinking. Previous studies showed that smoking and drinking are risk factors of high altitude illnesses [2], [11]–[12], and for people who going to altitude, tobacco and alcohol are strongly suggested to be avoided [3], [6], [8], [13]–[14]. Lindgärde and Lilljekvist (1984) found that smokers along with overconsumption of alcohol failed in long-term acclimatization when moving to high altitude, especially the moderate differences in hematocrit level (0.9) and hemoglobin concentration (0.2 g/100 ml) between male smokers and non-smokers at low altitude were almost quadrupled at 3,200 m height [15]. Wu et al. (2007) found that individuals who consumed large amount of alcohol were at a high risk of developing gastrointestinal bleeding [12]. Hansen and Claybaugh (1975) found that ethanol diminished respiratory gas exchange, causing lower alveolar and arterial oxygen pressures during normoxia and mild hypoxia and a reduction in arterial oxygen saturation from 89.9 to 87.4% during mild hypoxia [16]. Zapata-Ortiz et al. (1967) concluded that alcohol inhibits the initial stages of adequate acute ventilation adaptation to mild hypoxia at moderate altitude [17]. These studies mostly concentrated on demonstrating the effects of smoking or drinking on limited factors in respiratory function or hematology. As high altitude acclimatization is a series of physiological processes including ventilation function, cardiac function, oxygen delivery function, hematology and so on [5], the effect of individual factors, such as BMI, smoking and drinking, on these changes need to be further explored. And more importantly, the interaction effect between these individual factors and acclimatization time should be ascertained since high altitude acclimatization is a process significantly related with time.

In this study, a repeated measurement design based on three subsequent phases of high altitude acclimatization (baseline, acute phase, and chronic phase) was carried out on a sample of healthy Chinese young males. A series of physiological traits were collected on this sample at each phase of high altitude acclimatization. The aim of this study is to depict the physiological performances at acute phase and chronic phase of high altitude acclimatization taking baseline as background, and to evaluate the effect of BMI, smoking and drinking on these physiological changes at different phases of acclimatization.

## Materials and Methods

### Study design and background

We conducted a repeated measurement design to investigate the changes of physiological traits during high altitude acclimatization. The studied subjects were first assembled at a location with an altitude of ∼500 m for 10–14 days. And then they arrived at highland of above 3,500 m. The study is comprised of three phases: baseline (before going to highland), acute phase (AHA, immediately arrival at the highland), and chronic phase (CHA, living at highland for about 2 months). A structured questionnaire and physiological examination for the subjects were carried out at different phases of high altitude acclimatization, respectively. The subjects with major diseases (e.g., coronary heart disease, diabetes, cancer) were not included in this study. Overall 811 healthy Chinese Han young males aged from 16 to 22 years old were recruited. The research was approved by the Human Ethics Committee of Fudan University, and written informed consent was obtained from each participant and their guardians of 18 yrs old.

### Protocol and measurements

All the subjects were examined by physicians that were previously trained to administer a questionnaire and a physical examination. The age, sex, ethnicity, occupation, native place, altitude exposure, smoking and drinking behavior of each subject were collected. In our study, a smoker was defined as someone who smoked 1 or more cigarettes/day for >6 months. Non-smokers had never smoked. Smoking was classified as mild (<1 pack/day, i.e. 1–20 cigarettes/day) or heavy (> = 1 pack/day). A drinker was someone who drank >100 g alcohol each time and more than once per week. Non-drinkers had never drunk. Occasional smokers and drinkers were not included in the sample. All the subjects were asked to abstain from smoking and drinking when they first assembled due to their work requirements, so we could only evaluate the effect of smoking history and drinking history on physiological changes of high altitude acclimatization. For convenience of description, we still use smoking and drinking instead of smoking history and drinking history in following analysis.

Body weight and height were measured to calculate body mass index (BMI) (the body weight in kilograms divided by the square of height in meters). A standardized mercury sphygmomanometer was used to measure systolic blood pressures (SBP) and diastolic blood pressures (DBP). Heart rate (HR) was measured by mean of twice radial pulse. Forced expiratory volume at 1.0 second (FEV, L), maximum respiratory gas (PEF, L) and maximal vital capacity (FVC, L) were measured by SPIDA5 (Micro Medical Limited, Kent, ME1 2AZ, UK). Oxygen saturation (SaO_2_) was measured by Nellcor NPB-40 (USA). The blood specimens were drawn after overnight fasting for complete blood count measurement by SYSMEX (pocH-100i). The blood count traits include red blood cell count (RBC, ×10^12^/L), hemoglobin (HGB, g/L), hematocrit (HCT, %), mean corpuscular volume (MCV, fL), mean corpuscular hemoglobin (MCH, ps), mean corpuscular hemoglobin concentration (MCHC, g/L), white blood cell counts (WBC, ×10^9^/L), lymphocyte percentage (LYM%), intermediate cell percentage (MXD%), neutrophil percentage (NEUT%), absolute lymphocyte count (LYM), absolute intermediate cells count (MXD), absolute neutrophil count (NEUT), platelet count (PLT, ×10^9^/L), and standard deviation of red blood cell volume distribution width (RDWSD) and coefficient of variation of red blood cell volume distribution width (RDWCV).

### Statistical analysis

We described the demographical characteristics of the subjects in the results. We described the physiological responses of subjects from different altitude of native place at different phases of high altitude acclimatization. As the aim of this study is to evaluate the effect of BMI, smoking and drinking on a number of physiological changes at different phases of acclimatization, altitude of native place is a nonnegligible confounding factor. So we included the subjects from <2,000 m in our following analysis (755 subjects). Additionally, we explored the effect of BMI, smoking and drinking on each physiological trait during baseline, acute phase and chronic phase of high altitude acclimatization by considering altitude of native place as a confounding factor in mixed model. Mixed model analysis was done by mixed model procedure in SAS 9.1.3 [18]–[20].

As the relation among these physiological changes was complicated, we tried to extract composite phenotypes from the physiological traits. We applied Partial Least Square Path Model (PLSPM) to extract composite phenotypes from the physiological traits, and further evaluate the effect of BMI, smoking, and drinking on composite phenotypes during high altitude acclimatization by considering altitude of native place as a confounding factor. Before doing this, the longitudinal data was transformed to increment data,
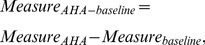
(1)

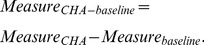
(2)


PLSPM was done by R language (package PLSPM0.1–11) [21]–[25].

## Results

### Demographic characteristics of the sample

**Figure 1:**
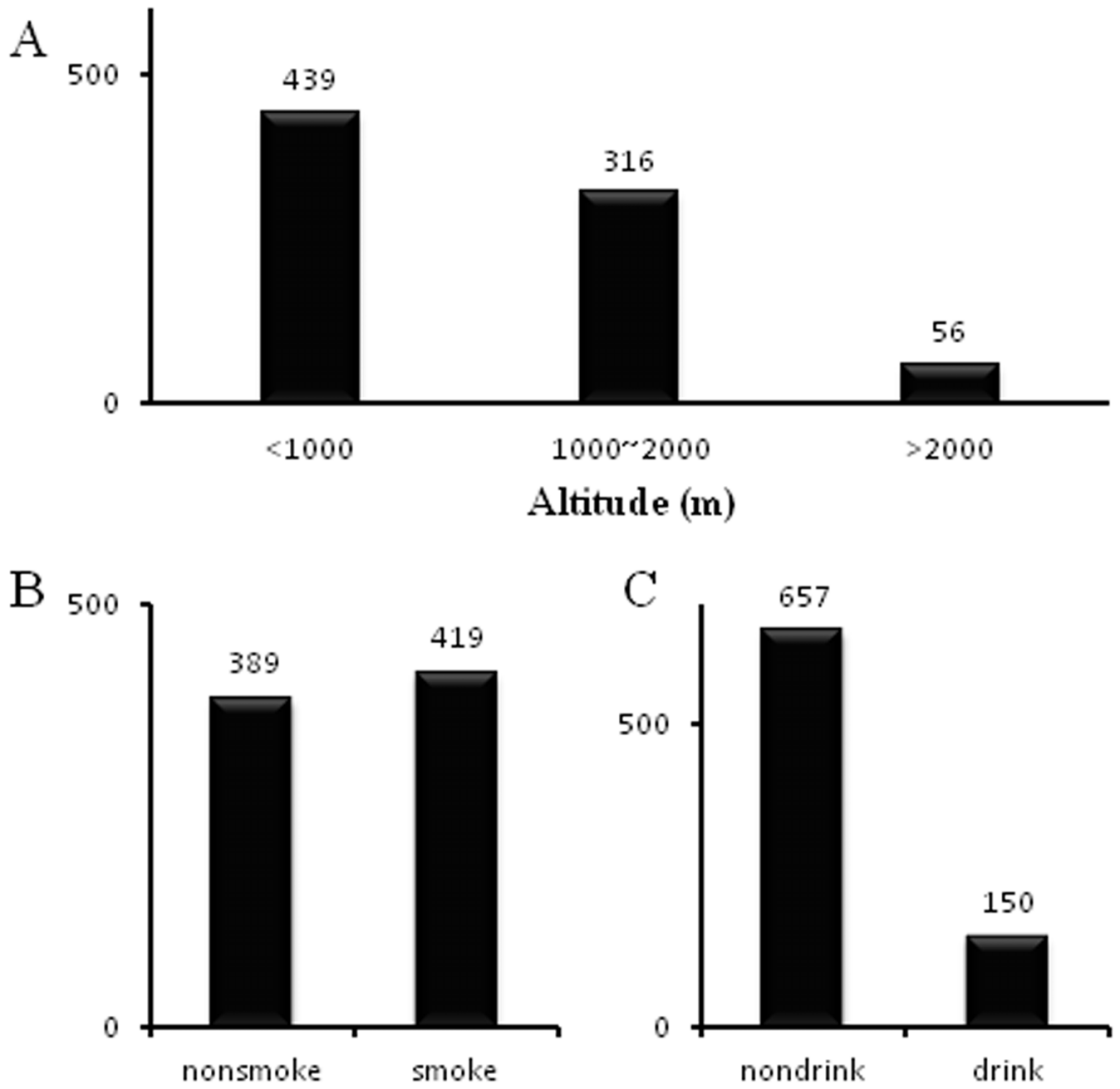
Demographic characteristics of individuals collected in the sample. A) The altitude distribution of subjects' native place; B) The smoking status of individuals; C) The drinking status of the individuals.

The study included 811 Chinese Han young males, with age of 16 to 22 years old (18.15±1.01) and BMI in normal range (20.61±2.05). None of the subjects carried major diseases (coronary heart disease, hypertension, diabetes, tumor and so on) and none of them developed severe high altitude illnesses. In our sample, 439 subjects came from <1,000 m, 316 subjects came from altitude 1,000 m to 2,000 m while 56 subjects came from altitude >2,000 m (see Figure 1A). Among the subjects, 389 individuals were nonsmokers, while the other 419 individuals were all mild smokers (Figure 1B). In the sample, 150 individuals in the sample were drinkers, and the other 657 individuals were nondrinkers (Figure 1C). Smoking duration and drinking duration ranged from 6 months to 3 years.

### Physiological responses of high altitude acclimatization

High altitude acclimatization includes a series of physiological traits which changes not only with time but also many other factors. We described the physiological performances of subjects from varied altitude of native place at different phases of acclimatization (Table 1), and found that the physiological performances of subjects from <2,000 m differed most significantly from those from >2,000 m (Table 1). At baseline, the SBP, MCH, MCHC and RDWCV of subjects came from altitude <2,000 m were lower than that of subjects from altitude >2,000 m, while HR, PEF, LYM%, LYM, MXD and PLT were higher than that of subjects from >2,000 m. At chronic phase, the MCHC, LYM and MXD of subjects came from <2,000 m were higher than that of subjects came from >2,000 m, while FEV, FVC and RDWCV were lower than that of subjects from >2,000 m. Furthermore, the trend of SBP, SaO_2_, FVC, MCH and MCHC performed differently during high altitude acclimatization for subjects from different altitudes. The SBP increased at acute phase and then decreased at chronic phase for subjects from <2,000 m, while it decreased at acute phase and then increased at chronic phase for subjects from >2,000 m. The SaO_2_ decreased at acute phase and then increased at chronic phase for subjects from <2,000 m, while it decreased at acute phase and then sustained at chronic phase for subjects from >2,000 m. The FVC decreased at acute phase and then increased at chronic phase for subjects from <2,000 m, while it increased chronologically for subjects from >2,000 m. The MCH increased chronologically for subjects from <2,000 m, while it increased at acute phase and then decreased at chronic phase for subjects from >2,000 m. The MCHC increased at acute phase and then decreased at chronic phase for subjects from <2,000 m, while it decreased straightly for subjects from >2,000 m.

**Table 1:** Physiological responses at different phases of high altitude acclimatization for individuals from different altitudes.

Trait	<1,000 m			1,000 m∼2,000 m			>2,000 m		
	baseline	AHA	CHA	baseline	AHA	CHA	baseline	AHA	CHA
**SBP**	101.18^1^	102.24	98.79	101.89	106.66	101.33	106.68^a^	101.04^b^	103.26
**DBP**	65.80	63.58	61.18	65.83	65.23	61.66	66.27	65.56	61.11
**HR**	80.10	101.38	90.07	80.99	99.03	86.96	70.41^a^	95.29	86.29
**SaO_2_**	98.50	86.66	91.20	98.37	88.04	91.14	98.75	91.27^b^	91.36^c^
**FEV**	3.70	3.78	3.92	3.75	3.85	3.95	3.71	3.92	4.07^c^
**PEF**	7.52	9.01	9.58	7.47	8.60	9.21	6.77^a^	8.66	9.08
**FVC**	4.32	4.28	4.42	4.60	4.53	4.67	4.41	4.60^b^	4.82^c^
**WBC**	6.65	7.21	7.42	7.27	7.15	6.99	6.11	6.21	6.82
**RBC**	4.82	5.05	5.16	5.07	5.15	5.04	4.75	5.06	5.09
**HGB**	140.08	149.99	153.36	152.20	155.59	153.33	145.30	155.63	152.91
**HCT**	0.41	0.43	0.45	0.44	0.44	0.45	0.41	0.44	0.45
**MCV**	84.52	85.64	87.61	86.41	86.36	88.56	86.88	87.69	89.38
**MCH**	29.21	29.76	29.80	30.19^a^	30.34	30.56	30.64^a^	30.78	30.03^c^
**MCHC**	345.47	347.50	339.77	349.21	351.26	344.91	352.60^a^	351.14^b^	336.22^c^
**PLT**	131.74	136.15	109.41	150.43	147.03	106.32	105.59^a^	113.33	102.13
**LYM%**	0.28	0.28	0.30	0.28	0.29	0.31	0.26^a^	0.30	0.29
**MXD%**	0.10	0.10	0.09	0.09	0.09	0.09	0.08	0.09	0.08
**NEUT%**	0.62	0.62	0.61	0.63	0.62	0.60	0.66	0.62	0.63
**LYM**	1.82	1.97	2.18	2.01	2.02	2.11	1.54^a^	1.79	1.90^c^
**MXD**	0.65	0.69	0.66	0.64	0.63	0.59	0.52^a^	0.51	0.56^c^
**NEUT**	4.21	4.54	4.61	4.64	4.52	4.26	4.04	3.90	4.36
**RDWCV**	40.63	42.12	45.86	41.29	40.95	45.59	42.31^a^	42.96	46.95^c^
**RDWSD**	0.13	0.14	0.14	0.13	0.13	0.14	0.13	0.14	0.14

1 mean of the physiological response.

a physiological responses performed differently at baseline phase of acclimatization (*α = 0.05*).

b physiological responses performed differently at acute phase of acclimatization (*α* = *0.05*).

c physiological responses performed differently at chronic phase of acclimatization (*α* = *0.05*).

As the aim of the study is to investigate the physiological changes of high altitude acclimatization when subjects going to high altitude from lowland area, altitude of native place is clearly a confounding factor. So we included the subjects from <2,000 m in our following analysis (755 subjects). Table 1 also showed that the physiological performances of WBC, RBC, HGB, HCT, PLT and LYM for subjects from 1,000 m–2,000 m were higher than those from <1,000 m during high altitude acclimatization, so altitude of native place was taken as a covariate in our study.

### Results of mixed model

We applied mixed model to evaluate the effect of BMI, smoking and drinking on each physiological trait at different phases of acclimatization. Taken SBP as an example (equation (3)), mixed model gives a regression function of SBP on an intercept (constant), altitude (covariate), BMI, smoking, drinking, acclimatization time, interaction between BMI and acclimatization time, interaction between smoking and acclimatization time and interaction between drinking and acclimatization time. Interaction between smoking and drinking was not significant on any physiological trait, so we did not include it in mixed model. At significance level of 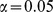, only the marginal effect of BMI, the interaction effect of BMI and acclimatization time (CHA, chronic phase), the interaction effect of drinking and acclimatization time (AHA, acute phase) were statistically significant. The marginal effect of BMI on SBP (coefficient *1.31, P<0.0001*) indicates that the mean SBP increases 1.31 unit for every increased unit of BMI after adjusting for other factors at baseline. The interaction effect of BMI and acclimatization time (BMI*CHA coefficient −*0.62, P = 0.02*) indicates that the mean SBP decreases 0.62 unit at chronic phase for every increased unit of BMI after adjusting for other factors. The interaction effect of drinking and acclimatization time (drinking*AHA coefficient *4.51, P = 0.003*) indicates that the mean SBP of drinking subjects increases 4.36 unit at acute phase after adjusting for other factors.
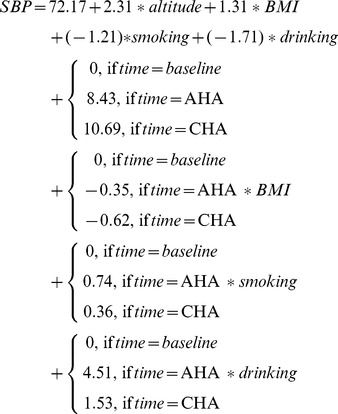
(3)


Table 2 listed the effect (coefficient) of BMI, smoking and drinking on each physiological trait at different phases of acclimatization obtained by mixed model. At baseline, the effect of BMI, smoking and drinking on most physiological traits were significant. At baseline, the SBP, DBP, FEV, PEF, FVC, WBC, PLT, NEUT and NEUT% were higher for subjects with higher BMI, while HR and LYM were lower. The FEV, MCV, MCH, MCHC and RDWCV of smoking subjects were higher than those nonsmokers, while DBP was lower. The PEF, FVC, WBC, RBC, HGB, HCT, PLT, LYM% and NEUT% of drinking subjects were higher than those nondrinkers, while SaO_2_, MCV, MCH, RDWCV and RDWSD were lower.

**Table 2:** The effect (coefficients) of BMI, smoking and drinking on physiological traits at different phases of high altitude acclimatization.

Trait	BMI			Smoking			Drinking		
	baseline	AHA	CHA	baseline	AHA	CHA	baseline	AHA	CHA
SBP	1.31±0.19*** a	−0.35±0.29	−0.62±0.27*	−1.21±0.81	0.74±1.21	0.36±1.12	−1.71±1.02	4.51±1.53**	1.53±1.41
DBP	0.73±0.15***	−0.38±0.21	−0.36±0.20	−1.29±0.63*	0.32±0.86	0.94±0.82	−0.35±0.80	0.91±1.08	−0.20±1.03
HR	−0.45±0.18*	0.39±0.30	−0.20±0.27	0.57±0.77	−1.31±1.24	−0.18±1.14	1.55±0.97	0.31±1.57	−4.18±1.43**
SaO_2_	−0.0004±0.0003	−0.003±0.0009**	−0.0004±0.0005	−0.0008±0.001	0.003±0.004	0.003±0.002	−0.003±0.001*	−0.01±0.005	0.01±0.003*
FEV	0.03±0.01***	−0.01±0.01	0.01±0.01	0.07±0.03*	−0.01±0.05	0.04±0.05	0.07±0.04	−0.01±0.06	−0.06±0.06
PEF	0.08±0.02**	0.002±0.03	0.002±0.03	−0.03±0.10	−0.003±0.14	0.02±0.14	0.41±0.12***	−0.33±0.17*	−0.21±0.18
FVC	0.07±0.01***	−0.01±0.01	−0.002±0.01	0.05±0.04	0.01±0.05	0.04±0.05	0.19±0.05***	−0.03±0.07	−0.11±0.07
WBC	0.08±0.03*	0.01±0.05	−0.09±0.05	0.02±0.13	−0.39±0.20	0.02±0.19	0.58±0.17***	0.18±0.26	−0.70±0.24**
RBC	0.01±0.01	0.02±0.02	0.02±0.01	0.02±0.05	−0.03±0.07	−0.003±0.06	0.42±0.06***	−0.21±0.08*	−0.49±0.07***
HGB	0.18±0.30	0.62±0.44	0.84±0.41*	2.22±1.25	−0.54±1.84	−0.45±1.71	10.69±1.58***	−6.69±2.31**	−13.36±2.15***
HCT	0.0005±0.0008	0.0008±0.001	0.001±0.001	0.01±0.003	−0.002±0.005	−0.001±0.005	0.03±0.004***	−0.02±0.01***	−0.04±0.01***
MCV	−0.03±0.08	−0.03±0.12	−0.10±0.11	0.89±0.34*	0.09±0.49	−0.32±0.48	−1.24±0.43**	−0.40±0.62	0.50±0.60
MCH	0.005±0.03	0.01±0.05	0.02±0.05	0.47±0.14**	−0.06±0.21	−0.19±0.20	−0.50±0.18*	0.01±0.26	0.31±0.26
MCHC	0.19±0.18	0.31±0.28	0.72±0.27*	1.88±0.75*	−1.08±1.18	−0.61±1.11	−0.73±0.95	1.86±1.49	1.45±1.40
PLT	3.25±0.73***	−1.02±1.02	−3.82±0.92***	−5.13±3.02	2.27±4.27	−1.83±3.81	15.61±3.80***	−13.77±5.36*	−11.85±4.79*
LYM	−0.003±0.001*	0.003±0.002	0.005±0.002*	0.01±0.01	0.002±0.01	−0.01±0.01	0.01±0.01	−0.01±0.01	0.01±0.01
MXD	−0.001±0.001	0.0002±0.001	−0.0001±0.001	0.004±0.004	−0.01±0.005	−0.01±0.005	−0.01±0.01	0.01±0.01	0.01±0.01
NEUT	0.004±0.002*	−0.002±0.002	−0.004±0.002*	−0.01±0.01	0.0009±0.01	0.01±0.01	−0.004±0.01	0.003±0.01	−0.01±0.01
LYM%	2.16E-03±0.01	0.02±0.02	0.01±0.02	0.08±0.04	−0.09±0.06	−0.05±0.07	0.22±0.06***	0.01±0.08	−0.14±0.08
MXD%	0.0008±0.00.	0.0002±0.01	−0.01±0.01	0.03±0.02	−0.08±0.03**	−0.04±0.03	0.01±0.02	0.06±0.04	−0.05±0.03
NEUT%	0.06±0.03*	−0.0001±0.04	−0.07±0.04	−0.04±0.11	−0.26±0.17	0.06±0.16	0.32±0.14*	0.14±0.21	−0.50±0.20*
RDWCV	−0.05±0.05	−0.02±0.07	−0.16±0.07*	0.67±0.21**	−0.17±0.30	−1.01±0.29***	−1.22±0.27***	−0.28±0.38	1.24±0.37***
RDWSD	−0.00009±0.0002	0.0001±0.0003	−0.0004±0.0002	0.0002±0.0008	0.0004±0.001	−0.002±0.001*	−0.003±0.001*	−0.001±0.002	0.004±0.001*

a coefficient and standard error.

*signicant under *α* = 0.05;

**significant under *α* = 0.01;

***significant under *α* = 0.001.

At acute phase, the effect of BMI on SaO_2_ was significantly negative (*−0.003, P = 0.0002*), which indicated that the SaO_2_ of subjects decreased along BMI at acute phase after adjusting for other factors. The effect of smoking on MXD% was significantly negative (*−0.08, P = 0.005*), which indicated that the MXD% of smoking subjects was lower than those of nonsmokers at acute phase. The effect of drinking on SBP was significantly positive (*4.51, P = 0.003*), while it on PEF, RBC, HGB, HCT and PLT were significantly negative (*−0.33, P = 0.05; −0.21, P = 0.01; −6.69, P = 0.004; −0.02, P = 0.0009; −13.77, P = 0.01*), which indicated that the SBP of drinking subjects was higher than those nondrinkers and their PEF, RBC, HGB, HCT and PLT were lower than nondrinkers at acute phase.

At chronic phase, the effect of BMI and drinking on most physiological traits were significant, while that of smoking were significant only on RDWCV and RDWSD. At chronic phase, the effect of BMI on SBP, PLT, NEUT and RDWCV was significantly negative (*−0.62, P = 0.02; −3.82, P = <0.0001; −0.004, P = 0.05; −0.16, P = 0.02*), which indicated that these physiological traits decreased with BMI at chronic phase. The effect of BMI on HGB, MCHC and LYM was significantly positive (*0.84, P = 0.04; 0.72, P = 0.007; 0.005, P = 0.02*), which indicated that these physiological traits increased with BMI at chronic phase. At chronic phase, the effect of smoking on RDWCV and RDWSD was significantly negative (*−1.01, P = 0.0006; −0.002, P = 0.04*), which indicated that the two physiological traits of smoking subjects were lower than that of nonsmokers. At chronic phase, the effect of drinking on HR, WBC, RBC, HGB, HCT, PLT and NEUT% was significantly negative (*−4.18, P = 0.004; −0.70, P = 0.004; −0.49, P<0.0001; −13.36, P<0.0001; −0.04, P<0.0001; −11.85, P = 0.01; −0.50, P = 0.02*), which indicated that these physiological traits of drinking subjects were lower than that of nondrinkers at chronic phase. The effect of drinking on SaO_2_, RDWCV and RDWSD was significantly positive (*0.01, P = 0.03; 1.24, P = 0.0008; 0.004, P = 0.005*), which indicated that these physiological traits of drinking subjects were higher than that of nondrinkers at chronic phase.

### Results of PLSPM

#### Composite phenotype

**Figure 2:**
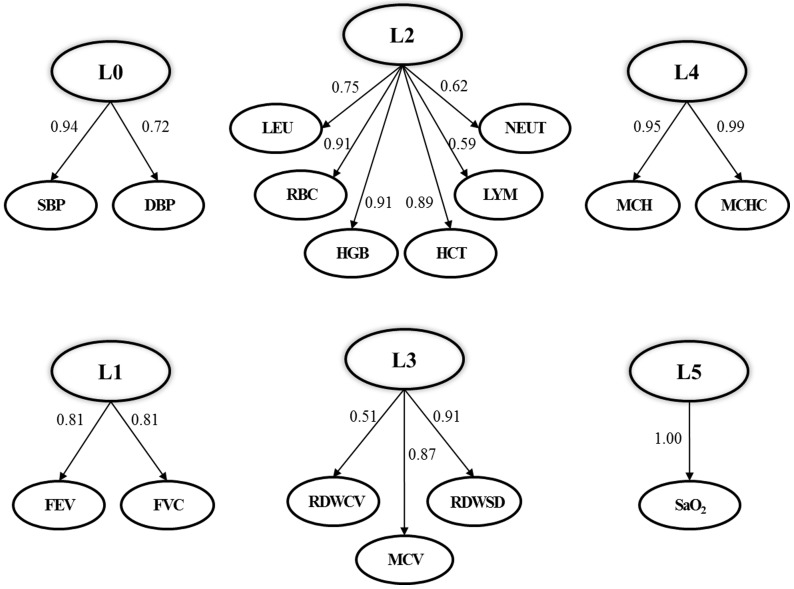
Composite phenotypes extracted from physiological traits by PLSPM. L0: blood pressure increment; L1: respiratory function increment; L2: blood cells counts increment; L3: red blood cell volume increment; L4: hemoglobin concentration increment; L5: oxygen saturation increment.

We extracted 6 composite phenotypes from the physiological traits collected in this study (see Figure 2), which were blood pressure changes extracted from manifest variables SBP and DBP (denoted as L0), respiratory function changes extracted from FEV and FVC (denoted as L1), blood cells counts changes extracted from WBC, RBC, HGB, HCT, LYM and NEUT (denoted as L2), red blood cell volume changes extracted from MCV, RDWSD and RDWCV (denoted as L3), relative hemoglobin changes extracted from MCH and MCHC (denoted as L4) and oxygen saturation (denoted as L5). Each composite phenotype explained a large proportion of the variation contained in the manifest variables. Taken blood pressure changes (L0) as an example, as shown in Figure 2, the loading coefficient (maximum = 1) on SBP and DBP were 0.94 and 0.72, which indicated that L0 explained the vast majority of changes of SBP and DBP.

#### Effect of BMI, smoking and drinking on composite phenotypes

We further evaluated the effect of BMI, smoking and drinking on extracted composite phenotypes at acute phase and chronic phase through PLSPM considering altitude of native place as a confounding factor. Bootstrap validation (1,000 times, confidence level 0.05) was conducted to screen reliable path coefficients among composite phenotypes and manifest variables[24]–[25].

**Figure 3:**
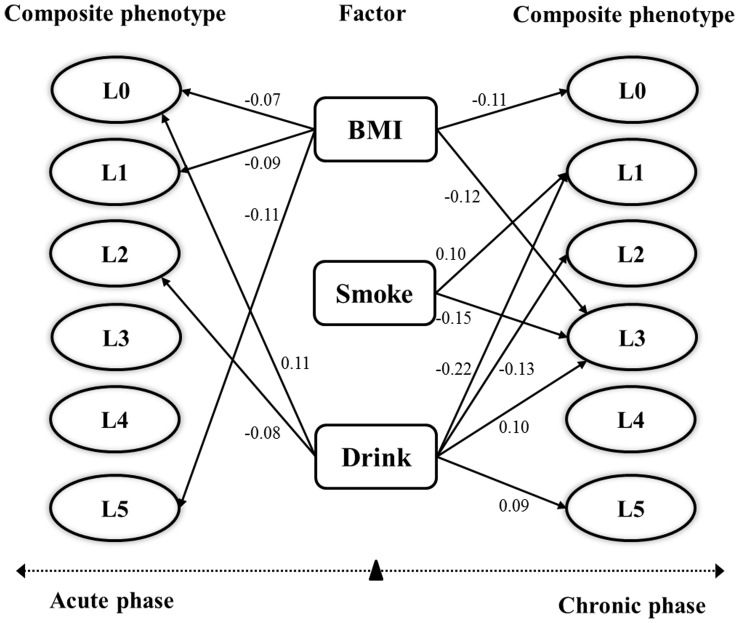
The effect (path coefficients) of BMI, smoking drinking on composite phenotypes evaluated by PLSPM and bootstrap at significance level (*α = 0.05*). L0: blood pressure increment; L1: respiratory function increment; L2: blood cells counts increment; L3: red blood cell volume increment; L4: hemoglobin concentration increment; L5: oxygen saturation increment.

At acute phase, shown in Figure 3, the effect of BMI on blood pressure changes (L0), respiratory function changes (L1) and oxygen saturation changes (L5) was significantly negative (path coefficient *−0.07, −0.09, −0.11*), which indicated that these composite phenotypes changes slower along with higher BMI. The effect of smoking was not significant on any composite phenotype at acute phase. The effect of drinking on blood pressure changes (L0) and blood cell counts changes (L2) was significant (path coefficient *0.11, −0.08*), which indicated that blood pressures of drinking subjects increased more while blood cell counts increased less than that of nondrinkers at acute phase.

At chronic phase, shown in Figure 3, the effect of BMI on blood pressure changes (L0) and red blood cell volume changes (L3) was significantly negative (path coefficient *−0.11, −0.12*), which indicated that the blood pressures and red blood cell volume changes slower along with higher BMI. The effect of smoking on respiratory function changes (L1) and red blood cell volume changes (L3) was significant (path coefficient *0.10, −0.15*), which indicated that respiratory function of smoking subjects increased more while red blood cell volume increases less than that of nonsmokers. The effect of drinking on respiratory function changes (L1), blood cell counts changes (L2) was significantly negative (path coefficient *−0.22, −0.13*) while it on red blood cell volume changes (L3) and oxygen saturation changes (L5) (path coefficient *0.10, 0.09*) was significantly positive, which indicated that respiratory function and blood cell counts of drinking subjects increased less than that of nondrinkers while red blood cell volume and oxygen saturation increased more than that of nondrinkers.

## Discussion

High altitude acclimatization is a series of physiological responses which takes place on subjects who go to high altitude, which has been demonstrated in comprehensive reviews [1]–[2], [26]–[27]. But how the physiological responses performed during high altitude acclimatization was still conflicting, as many factors could influence the physiological processes, such as altitude, temperature, climate, speed of ascending, physical status, psychical status, fluid taking and exercise.

Here, a study of repeated measurement design at three phases of high altitude acclimatization was conducted. The physiological performance before going to highland was taken as baseline. Acute phase was an important time point as many acute altitude illnesses occurred in this phase. The physiological, but not pathological, responses of the subjects would benefit our understanding of the mechanism of how the organism regulated itself to defend against the precipitate hypoxia environment and shepherd us in other studies related to hypoxia. Generally speaking, the organism could acclimatize to a new altitude in one to three months [2], [4]. In our study, the physiological performance at chronic phase could be taken as the consequence of high altitude acclimatization. In fact, at chronic phase, the organism had regulated its physiological performance to a new equilibrium acclimatizing to the hypoxia environment.

Another advantage of this study is the demographic characteristics of the sample. The subjects included in our study were the Chinese Han males aged 16–22 (18.15±1.01). The age, gender, occupation and other factors of the sample are homogeneous. And as the subjects included in the sample are young males, the physical status, psychical status, life style and exercise are controllable. These virtues of the sample ensured that there is little confounding effect implied in the data when we study the physiological processes of high altitude acclimatization.

Multiple traits association study is an important issue in complex trait study as a large proportion of complex traits are not independent traits. Although simple association study (e.g. mixed model in this paper) could detect the effect of factors on each trait, but how to precisely demonstrate the effect of factors on multiple dependent traits is still a difficult problem. In this study, a series of physiological responses were collected at three phases of high altitude acclimatization. From the results of mixed model, we discovered that some of the physiological traits tended to change in the same way according to influences of acclimatization time, BMI, smoking and drinking. We hypothesized that the physiological responses can be assembled into a few groups, and so we can evaluate the influences of factors on these grouped physiological responses. In this study, we applied PLSPM to extract composite phenotypes from multiple physiological responses and evaluate the effect of BMI, smoking and drinking on these composite phenotypes. The results of PLSPM helped us to understand the influences of factors on the process of high altitude acclimatization at system level.

### Physiological responses of high altitude acclimatization

Altitude acclimatization consists of physiological responses that develop in a time-dependent manner after arrival of high altitude [4]. At high altitude, the atmosphere pressure decreased abruptly, especially the partial pressure of oxygen decreased [4]. The oxygen inhaled and could be utilized by the organism thereupon decreased, in consequence, a series of physiological responses occurred to defend against oxygen deficit [2].

It was reported that acute hypoxia causes no change in the mean SBP [28], but in our study we found that SBP increased significantly at acute phase and returned to the baseline level at chronic phase for subjects from <2,000 m. The return of SBP could be taken as one of the indicators of acclimatization, as higher SBP accompanied with wider pulse pressure usually resulted in pathological changes [2]. The HR at chronic phase was higher than that at baseline, while the DBP was much lower than that at baseline. HR increased to maintain cardiac output [2], [28]. The cardiac function and blood circulation function was enhanced by increased HR and decreased DBP to a tolerable degree [2].

The increase in ventilation is one of the most important aspects of acclimatization [2]. At acute phase, respiratory function strengthened mostly depended on FEV and PEF, while at chronic phase FVC was reinforced for subjects from <2,000 m. The FVC decreased at acute phase, which might be the result of reduced ambient atmospheric pressure [4], [27], as the celiac baric pressure had not acclimatized to the precipitate reduction of ambient atmosphere pressure. At chronic phase, FVC was even higher than that at baseline.

The oxygen deficit also stimulates the bone marrow to increase red cell output[2]. We discovered that the blood cell counts increased significantly at acute phase and sustained at chronic phase, such as RBC, HGB and HCT, while PLT decreased significantly in order to benefit the blood flow [2]–[3], [29]–[31]. At chronic phase, the red blood cell volume continued to stretch and the MCH continued to increase, which enhanced the oxygen-carry capability of the blood. At acute phase, SaO_2_ decreased significantly, and at chronic phase after a series of physiological regulations, it rose again but still lower than that at baseline [4].

### Effect of BMI on physiological responses of high altitude acclimatization

Previous studies discovered that BMI indicated an inverse relationship with altitude in native population [9]–[10], [29], but there is scarce study focus on the relationship between BMI and high altitude acclimatization [2], [9]–[10]. In our study, we found that at baseline higher BMI was related with higher blood pressure (SBP and DBP), higher respiratory function (FEV, PFE and FVC), higher PLT and lower heart rate (HR).

The results of mixed model and PLSPM showed that the effect of BMI on physiological responses of high altitude acclimatization at acute phase and chronic phase were both negative, which indicates that subjects with higher BMI responses reluctant to hypoxia. At acute phase, blood pressure, respiratory function and oxygen saturation of subjects with higher BMI increased less than that of subjects with lower BMI. The reason for this phenomenon may be that there is scarce space for blood pressure or respiration function to increase at acute phase for subjects with higher BMI as they were already higher at baseline. At chronic phase, blood pressure and red blood cell volume of subjects with higher BMI increased less than that of subjects with lower BMI. As BMI is a risk factor for obesity [10] and obesity is a risk factor for high altitude illnesses [14], [32], higher BMI might be a risk signal of high attitude acclimatization.

### Effect of smoking on physiological responses of high altitude acclimatization

Earlier studies reported that red blood cell mass (RBC count and hematocrit [HCT]) and hemoglobin concentration increased in smokers [33]–[35]. In our study, we found that at baseline MCH, MCHC, MCV and RDWCV of smoking subjects was higher while DBP was lower than that of nonsmokers. At acute phase, the effect of smoking was not significant. At chronic phase, red blood cell volume of smoking subjects increased less while respiratory function increased more than that of nonsmokers. It is well-known that smokers are at high risk of suffering high altitude illnesses and are difficult to long-term acclimatization [14]–[15]. The result in our study only indicated limited effect of smoking on physiological responses of high altitude acclimatization.

### Effect of drinking on physiological responses of high altitude acclimatization

For drinking subjects, blood pressures increased more than nondrinkers at acute phase, red blood cell volume increased less than nondrinkers at both acute and chronic phase, and respiratory function increased less at chronic phase. In spite of this, red cell volume and oxygen saturation of drinking subjects increased more than nondrinkers at chronic phase.

At baseline, the respiratory function (PEF and FVC) of drinking subjects was higher than nondrinkers, which is in accordance to the discovery of Siu et al (2010) who reported that light to moderate drinkers of alcohol have better lung airway flow than alcohol abstainers [36]. We also found that blood cell counts (WBC, RBC, HGB, HCT and PLT) of drinking subjects were higher than nondrinkers, while their red blood cell volume (MCV, RDWCV and RDWSD) was lower than nondrinkers.

Klatsky et al. (1977) and Marmot et al. (1994) found that alcohol consumption was in positive relationship with blood pressures [37]–[38], but we did not discover this relationship at baseline. However, at acute phase, we found that the blood pressures (especially SBP) of drinking subjects increased more than nondrinkers. Ballard (1997) reported that heavy alcohol consumption caused generalized suppression of blood cell production and the production of structurally abnormal blood cell precursors that cannot mature into functional cells [13], it may be the reason of lower blood cell production in drinkers.

At chronic phase, drinking subjects relied on red blood cell volume increment (RDWCV and RSWSD) and oxygen saturation increment to maintain oxygen intake.

Smoking and drinking are frequently advised to abstain when subjects go to altitude [2], [4]. In our study, the effect of smoking and drinking on physiological processes of high altitude acclimatization was not very severe, which can be attributable to the following factors. First, the subjects in this study were young males (16 to 22 years old), and the smoking duration and drinking duration was only short term. Second, the status of smoking subjects and drinking subjects was mild. Third, when the subjects were first assembled, they were asked to abstain from smoking and drinking due to their work requirements, so we could only evaluate the effect of smoking history and drinking history on physiological responses of high altitude acclimatization. The effect of heavy and long-term smoking and drinking on physiological responses of high altitude acclimatization needed to be further studied.

### Limitations and perspective of this study

The study included 23 physiological traits, which covered respiratory function, cardiac function, oxygen delivery function, hematology, oxygen saturation and so on, but it was not sufficient. Other physiological traits, such as muscle structure and metabolism, oxygen consumption, organism metabolism, were not involved in this study. The subjects in this study were young males aged 16–22 years old, where the effect of some factor was incompletely exposed, such as smoking. We only evaluated the mild and short-term effect of smoking and drinking on physiological responses of high altitude acclimatization, the effect of heavy and long-term smoking and drinking needed to be further studied. Most importantly, other factors, such as genetic variations, may shed more light to the understanding of altitude acclimatization, which need to be studied.

## Conclusions

In this paper, based on a sample of 755 Han Chinese young males, we evaluated the effect of BMI, smoking and drinking on a number of physiological responses at different phases of high altitude acclimatization through simple association study (mixed model) and composite phenotype analysis (PLSPM). We found that subjects with higher BMI responses reluctant to hypoxia. The effect of smoking was not significant at acute phase. But at chronic phase, red blood cell volume increased less while respiratory function increased more for smoking subjects compared with nonsmokers. For drinking subjects, blood pressures increased more than nondrinkers at acute phase, respiratory function of drinking subjects increased less at chronic phase, and red blood cell volume increased less than nondrinkers at both acute and chronic phase. At chronic phase, red blood cell volume and oxygen saturation of drinking subjects increased more than nondrinkers.

## References

[1] Muza SR, Beidleman BA, Fulco CS (2010) Altitude preexposure recommendations for inducing acclimatization. High Alt Med Biol 11: 87–92. 20586592 10.1089/ham.2010.1006

[2] Ward MP, Milledge JS, West JB (2000) High Altitude Medicine and Physiology. New York: Oxford University Press.

[3] Curtis R (1998) High Altitude: Acclimatization and Illnesses.

[4] Muza SR, Fulco CS, Cymerman A (2004) Altitude Acclimatization Guide. ARMY RESEARCH INST OF ENVIRONMENTAL MEDICINE NATICK MA THERMAL AND MOUNTAIN MEDICINE DIVISION.

[5] Martin DS, Levett DZ, Grocott MP, Montgomery HE (2010) Variation in human performance in the hypoxic mountain environment. Exp Physiol 95: 463–470. 19946029 10.1113/expphysiol.2009.047589

[6] Gallagher SA, Hackett PH (2004) High-altitude illness. Emerg Med Clin North Am22: : 329–355, viii. 10.1016/j.emc.2004.02.001 15163571

[7] Fulco CS, Rock PB, Cymerman A (1998) Maximal and submaximal exercise performance at altitude. Aviat Space Environ Med 69: 793–801. 9715971

[8] Forgey WW (2006) High-altitude illness. Wilderness Medical Society: Practice Guidelines for Wilderness Emergency Care. New York. 46–53.

[9] Mandal C, Adak D, Biswas S, Bharati P (2011) A study on BMI among the Bhotia of Uttaranchal, India. Asian Pacific Journal of Tropical Disease 1: 55–58.

[10] Sherpa LY, Deji, Stigum H, Chongsuvivatwong V, Thelle DS, et al (2010) Obesity in Tibetans aged 30–70 living at different altitudes under the north and south faces of Mt. Everest. Int J Environ Res Public Health 7: 1670–1680. 20617052 10.3390/ijerph7041670 PMC2872340

[11] Wu TY, Ding SQ, Liu JL, Jia JH, Chai ZC, et al (2012) Who are more at risk for acute mountain sickness: a prospective study in Qinghai-Tibet railroad construction workers on Mt. Tanggula. Chin Med J (Engl) 125: 1393–1400. 22613641

[12] Wu TY, Ding SQ, Liu JL, Jia JH, Dai RC, et al (2007) High-altitude gastrointestinal bleeding: an observation in Qinghai-Tibetan railroad construction workers on Mountain Tanggula. World J Gastroenterol 13: 774–780. 17278202 10.3748/wjg.v13.i5.774 PMC4066012

[13] Ballard HS (1997) The Hematological Complications of Alcoholism. Alcohol Health and Research World 21: 42–52. 15706762 PMC6826798

[14] Wu TY, Ding SQ, Liu JL, Yu MT, Jia JH, et al (2007) Who should not go high: chronic disease and work at altitude during construction of the Qinghai-Tibet railroad. High Alt Med Biol 8: 88–107. 17584003 10.1089/ham.2007.1015

[15] Lindgarde F, Lilljekvist R (1984) Failure of long-term acclimatization in smokers moving to high altitude. Acta Med Scand 216: 317–322. 6496189 10.1111/j.0954-6820.1984.tb03810.x

[16] Hansen JE, Claybaugh JR (1975) Ethanol-induced lowering of arterial oxyhemoglobin saturation during hypoxia. Aviat Space Environ Med 46: 1123–1127. 1164347

[17] Zapata-Ortiz V, Batalla L, Gonzalez I (1967) Metabolism of alcohol in high altitudes. Acta Physiol Lat Am 17: 189–193. 5616494

[18] Verbeke G, Molenberghs G, editors (1997) Linear Mixed Models in Practice: A SAS-Oriented Approach. New York: Springer.

[19] Verbeke G, Molenberghs G (2000) Linear Mixed Models for Longitudinal Data. New York: Springer.

[20] Wolfinger RD (1997) An example of using mixed models and PROC MIXED for longitudinal data. J Biopharm Stat 7: 481–500. 9358325 10.1080/10543409708835203

[21] Michel Tenenhaus VEV, Chatelin Y, Lauro C (2005) PLS path modeling. Computational Statistics & Data Analysis 48: 159–205.

[22] Haenlein M, Kaplan AM (2004) A Beginner's Guide to Partial Least Squares Analysis. UNDERSTANDING STATISTICS 3: 283–297.

[23] Tenenhaus M (1998) La Regression PLS. Theorie et Pratique. TECHNIP, Paris.

[24] Chin WW (2003) A permutation procedure for multi-group comparison of PLS models. In: Vilares M., Tenenhaus M., Coelho P., Esposito Vinzi V., Morineau A., editor; Decisia. pp. 33–43.

[25] Chin WW (2000) Frequently Asked Questions, Partial Least Squares PLS-Graph.

[26] Young AJ, Reeves JT (2002) Human adaptation to high terrestrial altitude; Lounsbury DAE, Bellamy RF, Zajtchuk R, Washington, DC: Office of the Surgeon General, Borden Institute.

[27] Bisgard GE, Forster HV (1996) Ventilatory Responses to Acute and Chronic Hypoxia; Fregly MJ, Blatteis CM, New York: Oxford University Press.

[28] Kontos HA, Levasseur JE, Richardson DW, Mauck HP Jr, Patterson JL Jr (1967) Comparative circulatory responses to systemic hypoxia in man and in unanesthetized dog. J Appl Physiol 23: 381–386. 6069083 10.1152/jappl.1967.23.3.381

[29] Fiori G, Facchini F, Ismagulov O, Ismagulova A, Tarazona-Santos E, et al (2000) Lung volume, chest size, and hematological variation in low-, medium-, and high-altitude central Asian populations. Am J Phys Anthropol 113: 47–59. 10954619 10.1002/1096-8644(200009)113:1<47::AID-AJPA5>3.0.CO;2-K

[30] McDonald TP, Cottrell M, Clift R (1978) Effects of short-term hypoxia on platelet counts of mice. Blood 51: 165–175. 618555

[31] Srihirun S, Sriwantana T, Unchern S, Kittikool D, Noulsri E, et al (2012) Platelet inhibition by nitrite is dependent on erythrocytes and deoxygenation. PLoS One 7: e30380. 22276188 10.1371/journal.pone.0030380 PMC3262819

[32] Ge RL, Stone JA, Levine BD, Babb TG (2005) Exaggerated respiratory chemosensitivity and association with SaO2 level at 3568 m in obesity. Respir Physiol Neurobiol 146: 47–54. 15733778 10.1016/j.resp.2004.11.009

[33] Nordenberg D, Yip R, Binkin NJ (1990) The effect of cigarette smoking on hemoglobin levels and anemia screening. JAMA 264: 1556–1559. 2395196

[34] Tarazi IS, Sirdah MM, El Jeadi H, Al Haddad RM (2008) Does cigarette smoking affect the diagnostic reliability of hemoglobin alpha 2 delta 2 (HbA2)? J Clin Lab Anal 22: 119–122. 18348310 10.1002/jcla.20228 PMC6648953

[35] Myersa SR, Spinnatoa JA, Pinorini-Godlya MT (2000) Tobacco Smoke Hemoglobin Adducts in Maternal and Fetal Blood. Polycyclic Aromatic Compounds 21: 151–166.

[36] Siu ST, Udaltsova N, Iribarren C, Klatsky AL (2010) Alcohol and Lung Airways Function. Perm J 14: 11–18. 10.7812/tpp/09-089 PMC2912709 20740126

[37] Klatsky AL, Friedman GD, Siegelaub AB, Gerard MJ (1977) Alcohol consumption and blood pressure Kaiser-Permanente Multiphasic Health Examination data. N Engl J Med 296: 1194–1200. 854058 10.1056/NEJM197705262962103

[38] Marmot MG, Elliott P, Shipley MJ, Dyer AR, Ueshima H, et al (1994) Alcohol and blood pressure: the INTERSALT study. BMJ 308: 1263–1267. 7802765 10.1136/bmj.308.6939.1263 PMC2540174

